# Lactoferrin for Prevention and Treatment of Anemia and Inflammation in Pregnant Women: A Comprehensive Review

**DOI:** 10.3390/biomedicines9080898

**Published:** 2021-07-27

**Authors:** Jolanta Artym, Michał Zimecki, Marian L. Kruzel

**Affiliations:** 1 Department of Experimental Therapy, Hirszfeld Institute of Immunology and Experimental Therapy, Polish Academy of Sciences, R. Weigla 12 Str., 53-112 Wrocław, Poland; jolanta.artym@hirszfeld.pl; 2 Department of Integrative Biology and Pharmacology, McGovern Medical School at Houston, University of Texas, 25 7505 Fannin Str., Third Floor 313, Houston, TX 77054, USA; Marian.L.Kruzel@uth.tmc.edu

**Keywords:** lactoferrin, pregnancy, iron deficiency, iron deficiency anemia, anemia of inflammation

## Abstract

Pregnancy is a physiological state that demands higher level of nutrients, including vitamins and minerals, for the growth and maintenance of the fetus. Iron deficiency is a part of most common diet deficiencies in pregnancy and has high clinical significance leading to the development of syderopenic anemia and its consequences for mother and child, such as higher risk of perinatal death, susceptibility to infection, intra-uteral growth inhibition, prematurity and low birth weight. Hence, iron supplementation is recommended for pregnant women; however dietary intake of iron from most commercially available formulas is often insufficient due to iron-poor bioavailability, or have undesired side-effects in the gastrointestinal tract, resulting in a discouraging and distrustful attitude to such treatment. The results of numerous studies indicate that diet supplementation with lactoferrin (LTF), an iron-binding protein, may be advantageous in prophylaxis and treatment of iron deficiency anemia. LTF, administered orally, normalizes iron homeostasis, not only by facilitating iron absorption, but also by inhibiting inflammatory processes responsible for anemia of chronic diseases, characterized by a functional iron deficit for physiological processes. LTF also protects against infections and inflammatory complications, caused by diagnostic surgical interventions in pregnant women. Beneficial, multidirectional actions of LTF during pregnancy encompass, in addition, inhibition of oxidative stress, normalization of intestine and genital tract microbiota and carbohydrate-lipid metabolism, protection of intestine barrier function, promotion of wound healing, as well as hypotensive, analgesic and antistress actions. Bovine lactoferrin (BLTF) is readily available on the nutritional market and generally recognized as safe (GRAS) for use in human diet.

## 1. Introduction

Pregnancy is associated with major physiological changes which ensure the best environmental conditions for embryo and fetus for growth and development. During pregnancy, the demand for energy and nutrients is increased, including vitamins, micro and macro elements. Consequently, daily supplementation in pregnant women’s diet is recommended to compensate for insufficient intake of these nutrients from regular diet. In pregnant women most frequent deficiencies pertain to iron, folic acid, calcium, iodine, and vitamins A and D. According to the World Health Organization (WHO), deficiency of iron and folic acid is of clinical importance, often leading to dangerous physiological imbalance, such as anemia, and is common both in developing as well as industrialized countries [1]. In 2011, worldwide 19.2% of pregnant women had anemia attributed to iron deficiency (global prevalence of overall anemia for pregnant women was 38.2%) [2,3]. Evidence also exists that iron deficiency anemia (IDA) in pregnancy is associated with increased maternal morbidity and mortality as well as an increased risk of adverse outcomes in the offspring (preterm birth, low birth weight, neurobehavioral defects) [4,5].

Due to the major clinical significance of nutrient deficiencies, their supplementation in the diet is highly recommended, yet needs to be carefully crafted. Oral iron supply (in iron-rich food and iron supplements) is the first choice to replete body iron stores. Most frequently used non-heme iron formulas such as iron sulfate, fumarate and gluconate given orally are moderately (20%–30%) bioavailable, hence they are administered at high doses causing numerous, strong, undesirable effects, associated mainly with gastrointestinal disorders (loss of appetite, abdominal pain, nausea, vomiting, diarrhea, constipation) which makes compliance even worse. Gastrointestinal distress is due to unabsorbed iron from the supplements that may promote oxidative stress, subclinical inflammation and undesirable shift in gut microbiome profile. Exacerbation of oxidative stress and inflammatory states in pregnancy, due to application of elemental iron, especially when given in high doses (orally or parenterally), may also increase risk of serious systemic events such as premature delivery, preeclampsia, glucose intolerance/gestational diabetes, low birth weight and cognitive defects in newborns [1,6,7,8,9,10,11,12].

Therefore, a supplementation with iron needs to be carefully balanced, as both too much iron and insufficient supplementation may increase the risk for pregnant woman and fetus. Accordingly, novel clinical protocols for safe prophylaxis and treatment of anemia in pregnant woman are needed. Easily accessible and safe non-heme organic iron sources can be found among naturally iron-binding proteins, such as plant ferritin and mammalian lactoferrin (lactotransferrin, LTF) [13,14]. LTF is an especially promising alternative to inorganic iron formulas currently used for the prophylaxis and treatment of anemia in pregnant woman. As a multifunctional immune modulator, antioxidant agent and regulator of intestinal iron absorption, LTF has already been shown to be effective, with a safety profile for prophylaxis and treatment of some anemia and chronic inflammation in pregnant women.

In this article we review some of the pre-clinical and clinical studies on LTF, taking into consideration plausible regulatory mechanisms of its action, as well as practical aspects of its application as a diet supplement in pregnant women with IDA, both in healthy pregnancy and pregnancy affected by infections and inflammations of various etiology.

## 2. The Role of Iron and Its Metabolism in Pregnancy

Iron (lat. ferrum, Fe) belongs to the dietary microelements. The average daily intake of iron from foods and supplements is 15–20 mg depending on age, gender, and health condition in humans. Iron is essential for the maintenance of many physiological processes in our body [15,16]. Iron may be present as reduced ferrous ions (Fe^2+^) or oxidized ferric ions (Fe^3+^) while being at the same time electron acceptor and donor (Fe^2+^ ⇋ Fe^3+^). For this reason, iron participates in numerous metabolic processes. It is responsible for transport of oxygen in erythrocytes, generation of energy in mitochondria, synthesis and DNA repair, synthesis of thyroid hormones, transmission of nerve impulses, tightness of blood–brain barrier, regulation of lipid metabolism and many other cellular events. In addition, iron conditions competent action of the immune system (among others the activity of B, T and NK cells) ensuring protection against infection and cancer development. Of importance, it participates in oxidation-reduction processes, generating among others: superoxide (O_2_^•^), hydrogen peroxide (H_2_O_2_), and hydroxyl radical (OH^•^), collectively termed reactive oxygen species (ROS). In the physiologic state ROS play a role in defense against pathogenic bacteria and fungi as well as in cellular signaling. An excessive ROS production, however, is not neutralized by the body’s anti-oxidative protective systems resulting in oxidative stress during which these reactive species may be toxic for hosts’ cells leading to the establishment of chronic inflammatory conditions.

**Figure 1:**
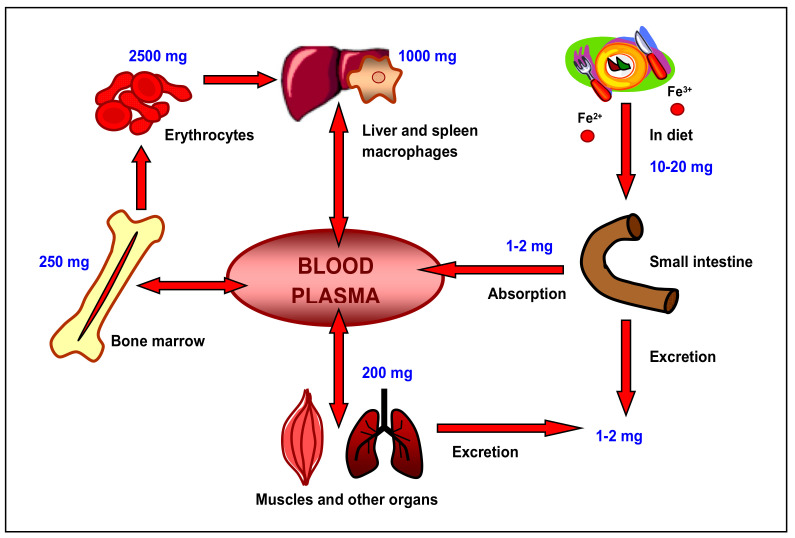
Turnover of iron in the human body. Given values indicate amounts of absorbed, used, stored and expelled iron.

On average adults accumulate about 4 g of iron (45 mg/kg b.w.), mostly contained in the hemoglobin of circulating erythrocytes (60–70%), 20%–30% in storage proteins (hepatic and splenic ferritin and hemosiderin), 10% in myoglobin, cytochromes and enzymes and 1% (about 4 mg) associated with a transport protein—transferrin (TF). A daily uptake of dietary iron corresponds to about 1–2 mg, and a similar amount is excreted, mainly with desquamated epithelia. For metabolic processes iron is repeatedly utilized from disintegrated old erythrocytes in the liver and spleen, then its metabolism runs almost in a closed circuit. The utilization of iron in the body is schematically presented in Figure 1.

**Figure 2:**
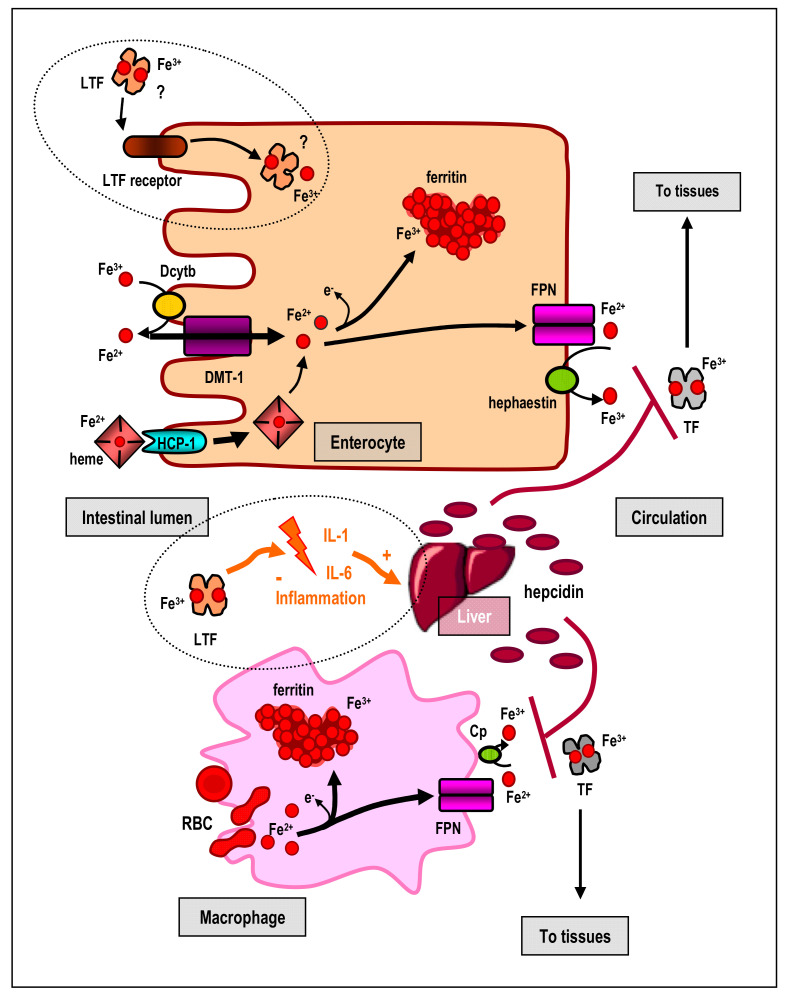
Processes of iron absorption in small intestine enterocytes and release from hepatic/spleen macrophages. For uptake of non heme Fe^3+^ ions, their reduction to Fe^2+^ is essential, involving Dcytb. Fe^2+^ ions are transported into cells by means of DMT-1 transporter and here may be stored in a form of ferritin (after prior oxidation) or released into circulation by FPN transporter (after prior oxidation by membrane hephaestin or serum Cp). In circulation, Fe^3+^ ions bind to TF that transports them to all tissues; in the left upper corner (in the circle) a possible participation of LTF in the process of iron absorption is presented. Iron from waste, recycled erythrocytes, is accumulated in hepatic/splenic macrophages (reticuloendothelial system) as ferritin or, as necessary, released from cells by means of FPN, bound to TF and transported into tissues. In both cases, hepatic hepcidin (induced among other by proinflammatory cytokines) inhibits activity of FPN and iron release into circulation. LTF inhibits expression of proinflammatory cytokines and in this way inhibits expression of hepcidin, ensuring activity of FPN and iron absorption and its release from body resources (in the middle of figure, in the circle). Cp–ceruloplasmin, Dcytb–duodenal cytochrome b, DMT-1–divalent metal transporter 1, Fe–iron ions, FPN–ferroportin, HCP-1–heme carrier protein 1, IL–interleukin, LTF–lactoferrin, RBC–red blood cells, TF–transferrin.

No particular system of iron excessive excretion exists; hence its storage levels are regulated at the level of iron uptake. Iron ions are acquired from the diet by enterocytes of duodenum and jejunum [14,17,18] and transported by carrier protein—divalent metal transporter 1 (DMT-1). Enterocytes are penetrated only by Fe^2+^ ions. Diet contains also Fe^3+^ ions, which before absorption are reduced to Fe^2+^ ions by the action of duodenal cytochrome b (Dcytb), adjacent to DMT-1 in the brush border membrane. Heme Fe^2+^ ions (contained in meat) are transported to enterocytes via heme carrier protein 1 (HCP-1) and released in the cytosol by heme oxygenase 1 (HO-1). In enterocytes Fe^2+^ ions are transiently stored in iron-binding protein—ferritin (Ftn), or released into circulation via basolateral membrane transporter—ferroportin (FPN), the only known exporter of iron ions from cells. FPN is expressed in all cells that export iron ions into plasma: enterocytes, macrophages and placental syncytio-trophoblast. Fe^2+^ ions are oxidized to Fe^3+^ ions by action of oxidases: membrane hephaestin or serum ceruloplasmin (Cp). In the blood Fe^3+^ ions are transported by TF molecules to all cells and tissues including bone marrow to support erythropoiesis. The processes of iron absorption in the intestine and release from hepatic or splenic macrophages are presented in Figure 2.

Expression of both iron transporters, DMT-1 and FPN, are regulated by systemic iron resources: when there is a lot of iron this decreases, and increases again when reserves are empty. Additionally, at the systemic level, iron homeostasis is regulated by a small hepatic hormone protein—hepcidin, which acts as a master regulator of iron. Hepcidin, by binding to FPN, triggers internalization and degradation of this membrane transporter and inhibits release of iron from the gut into circulation. Likewise, release of iron from the reservoirs in hepatic and splenic macrophages is also blocked. Several factors may affect synthesis of hepcidin, such as availability of iron, intensity of erythropoiesis, tissue hypoxia and presence of proinflammatory molecules [17,19,20,21].

Fetus acquires iron from mother via placenta, by means of TF, TF receptor (TFR), DMT-1 and FPN in the placental syncytio-trophoblast cells. It can also synthesize in the liver its own hepcidin which may regulate placental iron transport which is augmented in cases of maternal iron deficit. Fetal signals in placental iron transport are incompletely understood and are under extensive research. However, as was indicated in the latest animal and human studies, amniotic and fetal plasma iron during infection and inflammation is regulated by fetal hepcidin [22]. The iron flow is unidirectional—always directed from mother to fetus (never from fetus to the mother), and fetus’ needs are superior and competitive with respect to mother [23]. Eighty per cent (80%) of iron storage is acquired by the fetus during the last trimester and this pool may be significantly enlarged by 40–500 mg iron/kg body weight by a delay in umbilical cord clamping at birth for an additional 1–3 min for blood transfusion from the placenta to the newborn. The newborn at term born to nonanemic iron-sufficient women has iron stores for disposal during the first 4–6 months which is significant in cases of physiologically low iron content in mother’s milk [5,24,25]. Noteworthy is that for many years the fetus was treated as a “perfect parasite” that is able to acquire sufficient iron even from a mildly/moderately anemic mother. At present it is known that the paradigm is not correct. The data available now confirm that, in spite of iron being prioritized to the fetus, in moderately/severely anemic mother iron transport to fetus becomes insufficient and suboptimal fetal/neonatal iron stores may lead to long-term, irreversible metabolic and developmental impairments, particularly in the developing brain, a highly metabolic organ [4,5,24]. Other gestational conditions (in addition to maternal iron deficiency) considered as risk factors for low fetal and neonatal iron status are: premature delivery, fetal growth restriction due to maternal undernutrition and hypertension (including preeclampsia), maternal smoking during pregnancy and pregestational and gestational diabetes [4].

## 3. Demand for Iron and Iron Deficiency in Pregnancy

The recommended dietary allowance (RDA) for daily iron intake by non-pregnant woman in the reproductive period has been established in accordance with directions from The Institute of Medicine (IOM, Washington, DC, USA), at 18 mg as iron combined from regular diet and diet supplements [26,27]. Nevertheless, only 10% (about 1.8 mg) from this amount is absorbed. Heme derived iron (Fe^2+^), contained in red meat, poultry and fish, is better assimilated (25%) than non heme iron (Fe^3+^) prevailing in diet (vegetable products, eggs, milk), with only 5% absorbed [28]. During pregnancy, the requirement for iron increases two-fold due to the development of placenta, fetus and maternal tissues, so the increasing hemoglobin mass is established at 27 mg (RDA). This demand for iron is covered by daily intestinal, physiological iron absorption at 3–4 mg/day in the first and 8–9 mg/day in the third trimester. The total amount of iron used in pregnancy corresponds to about 1000–1200 mg, from which 500–600 mg is utilized for the sake of pregnancy development (nearly 300 mg is deposited in the fetus) and the rest for basic needs of the mother [5,26,27,28]. In healthy pregnancy physiological down regulation of maternal serum hepcidin is observed to enhance intestinal iron absorption and its release from stores in spleen and liver and transport to the fetus. The reset in maternal hepcidin level is essential for maternal and fetal iron homeostasis and provides strong evidence for high iron need during pregnancy. Mechanism of maternal hepcidin suppression in pregnancy and re-increase immediately after delivery is hitherto unknown [10,23].

Well-balanced and differentiated diet is the best source of iron. However, the majority of women do not modify their dietary habits before and after conception so that requirement for iron is not covered. It is also hard to meet the needs of vegetarians or vegans because of weak assimilation of vegetable non-heme iron and a dairy product diet that restricts iron absorption. Lack of appetite, nausea and vomiting also create problems. Pregnant women with disturbances in iron absorption and greater demand for supplies e.g., with chronic gut inflammation or celiac disease, stomach erosion/ulceration due to *Helicobacter pylori* infection and non-steroid anti-inflammatory drug (NSAID) treatment, chronic bleeding from hemorrhoids, as well as women with gestational diabetes (due to abnormal function of placental TFR), multiparous with short period intervals between pregnancies, and pregnant adolescents, are also in danger [24,27].

Studies conducted in 14 European countries (between 1991 and 2014) reveal that as much as 60–100% of pregnant women acquire via diet lower amounts of iron than is recommended (in Poland almost 100%) [26]. The situation becomes even worse since 40% of European women in the reproductive period experience lack of or low systemic iron storage, as reflected by low serum ferritin levels (sFtn < 30 µg/L; every 1 µg of circulating Ftn represents 10 mg of stored iron) [24,26]. Thus, in these instances a state of iron deficiency (ID) develops that may lead to IDA, also known as syderopenic anemia and characterized by hypochromic and microcytic red blood cells. ID is a non-anemic state albeit with a negative iron balance, where iron supply does not provide for iron tissue demand. In ID iron-dependent processes in every cell and organ, such as energy metabolism, cellular gene expression, thyroid hormone synthesis, neuronal growth and differentiation and myelination, are compromised, so the detrimental effects of iron deficiency are particularly profound during development [4]. Iron is prioritized for hemoglobin (Hb) synthesis in erythrocytes so IDA is the end stage of body iron deficiency [5].

As the Center for Diseases Control (CDC) recommended, IDA is diagnosed when serum Hb concentration is below 11 g/dL in I and III trimester and below 10.5 g/dL in II trimester [1]. The anemia is threatening since it increases risk of complications both for pregnant woman (increased risk of bleeding at delivery, perinatal death and susceptibility to infection), and for fetuses/newborns (intra-uteral growth inhibition, prematurity, low birth weight, anemia, susceptibility to infection, neurodevelopmental impairment) [5,29]. A particular interest is paid to inferior psychomotor development of anemic mothers’ progeny, for iron is indispensable for growth of all tissues and organs, in particular the fetal brain, including limbic system and hippocampus, responsible for emotion, memory and learning. Of importance, neurobehavioral abnormalities (deficiency in motor, emotional and social development, increased risk of autism, schizophrenia and mood disorders) due to iron deficit in the perinatal period are permanent and irreversible despite postnatal iron repletion [4,5,23,25].

It is estimated that IDA affects as much as 43% of the world’s (45 million) and 25% of European (2.5 million) pregnant woman population. In USA and Germany ID is found in 18% and 40% of pregnant women, respectively, and in Denmark 50% and 21% of women suffers from ID and IDA, respectively [1]. In 85% of pregnant Polish women the iron stores of the organism are exhausted by the end of pregnancy and almost half of them develop IDA, predominantly (80% of women) due to iron deficit and subsequently to shortages of folic acid (vitamin B9) and cobalamin (vitamin B12) [27]. These vitamins participate in metabolic processes including erythropoiesis, hence they should also be included in iron supplementation. Vitamin D is a negative regulator of inflammation and hepcidin expression (so its deficiency increases hepcidin formation and iron retention), therefore its supplementation is also worth considering [21].

The development of ID/IDA during pregnancy is further related to coexisting, chronic inflammatory states. A sterile, moderate physiological inflammation accompanying pregnancy is normal, particularly in the first and third trimester, conditioning its proper development and delivery [30,31]. However, it is frequently intensified due to ongoing infection/inflammation (especially chronic, as malaria) or so-called metabolic endotoxemia occurring in metabolic syndrome (usually in obese women with type II diabetes and dyslipidemia) [4,15,32]. Data from the clinical observation found that obesity in pregnant women was associated with decreased maternal iron status, possibly due to increased level of maternal hepcidin and that compensated for by increased expression of placental TFR [33].

Inflammation is an important factor in controlling iron metabolism via proinflammatory cytokines released by activated immune cells. Interleukin (IL)-1, IL-6, IL-22 and interferons (IFNs) induce secretion of hepcidin that blocks absorption of dietary iron and its release from reticuloendothelial deposits in liver and spleen [17,20,21]. Such a functional state of iron deficit (not true iron deficiency) protects against growth of pathogens and oxidative stress and is described as anemia of inflammation (AI) or anemia of chronic diseases (ACD). It is, among others, characterized by the following iron status and inflammation indices: elevated serum hepcidin concentration, low level of total serum iron (TSI) and Hb (<11 g/dL), normal/enhanced sFtn level (>100 μg/L), high level of C-reactive protein (CRP), erythrocyte sedimentation rate (ESR) and IL-6. Contrary to IDA, AI is moderate anemia characterized by normochromic and normocytic erythrocytes [5,21,24]. Elevated hepcidin in inflammation is especially dangerous in pregnancy because it may limit iron availability to placenta and fetus.

**Figure 3:**
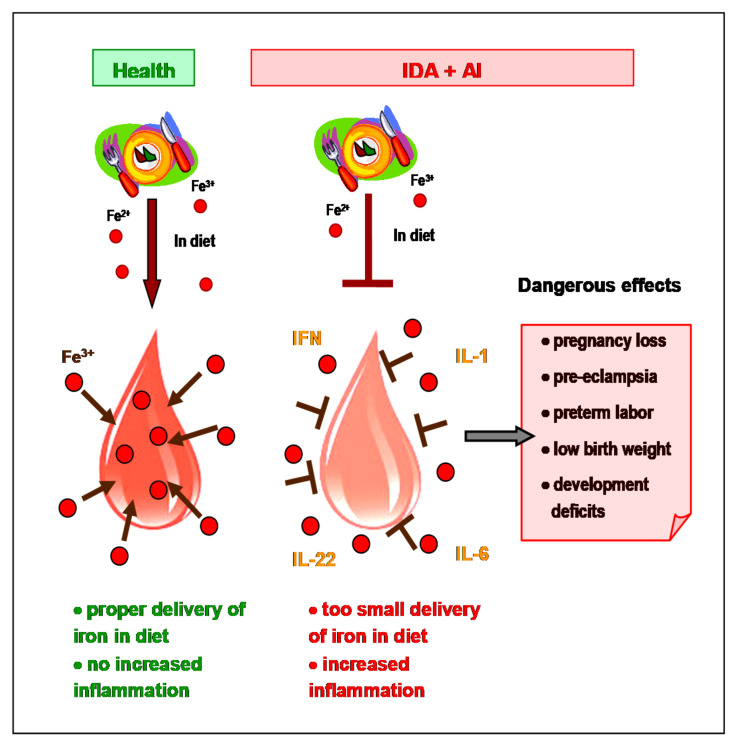
Dangerous consequences of coexisting iron deficiency anemia (IDA) and anemia of inflammation (AI) during pregnancy. Inadequate delivery of iron from diet is responsible for “true” deficit (IDA), which is aggravated by simultaneous inflammation responsible for “functional” deficiency (AI). IDA and AI are dangerous to mother and her offspring. Fe—iron ions, IL—interleukin, IFN—interferon.

Pregnant woman with functional iron deficiency (AI) and concomitant true iron deficiency (IDA), described as AI/IDA patients are at particular risk, because of elevated requirements for additional iron. Closely related AI and IDA represent independent threat factors for pregnancy, preterm delivery, perinatal woman or newborn death and postnatal child development (Figure 3). Moderate inflammation in normal pregnancy has no effect on increase of serum hepcidin, but hepcidin, significantly elevated due to exacerbated inflammation in complicated (inflamed) pregnancy, may impair body iron stores mobilization and intestinal iron absorption from food and prescribed supplements/drugs and even reduce efficacy of parenteral iron therapy [23]. Then, combating the excessive inflammatory state is of primary importance in this complicated and dangerous situation, followed by appropriate iron supplementation.

## 4. Iron Supplementation during Pregnancy

Presently, there is no definite consensus on iron supplementation during pregnancy. The WHO, in its latest report on global occurrence of anemia in 2011, recommends daily iron supplementation at 30–60 mg for all pregnant women and those planning pregnancy to diminish the risk of complications for the mother and child [3]. Similarly, CDC, Food and Agricultural Organization of the United Nations (FAO) and Nordic Nutrition Recommendations (NNR) conclude that diet cannot ensure an appropriate supply of iron and recommend its supplementation during pregnancy [26]. However, the majority of scientific associations with a focus on pregnant woman, such as the Royal College of Obstetrics and Gynaecology (RCOG), the Royal Australian and New Zealand College of Obstetrics and Gynaecology (RANZCOG), the British Society for Haematology, the Obstetric Haematology Group (BSH OHG), British Committee for Standards in Haematology (BCSH), the Scientific Advisory Committee on Nutrition (SACN) and the European Food Safety Authority (EFSA) do not share this opinion and advise iron supplementation in pregnancy only in cases of its deficit. The controversy arises from difficulties in the final assessment of potential benefits and risks of routine iron supplementation in pregnancy [4,12].

Taking into consideration undesirable consequences of both iron deficit and its excess, as well as other causes of anemia, such as infection, inflammation and B12 and/or folic acid deficits, the health authorities of many countries including the Polish Society of Gynecologists and Obstetricians did not implement the WHO recommendation [12]. Accordingly, earlier recommendations (before 2020) for the Polish population advised iron supplementation prior to planned pregnancy, with an eight-week interval after conception, because of developmental risk of defects if iron concentration in the follicular fluid is too high, and its continuation to delivery or even during lactation. A prophylactic, low dose (30 mg/day) and therapeutic dose (80–120 mg/day) for women with confirmed IDA was recommended [27,34]. Present guidelines, on the other hand, recommend iron supplementation only in women with laboratory confirmed IDA, i.e., with diagnosed lowered Hb level, mean corpuscular volume (MCV) and sFtn level. Normal ferritin levels suggest the presence of anemia of another etiology (AI or with deficit of B12/folic acid). Ferritin content of 60–70 µg/L indicates sufficient body iron stores and there is no need for supplementation (low risk of IDA). At ferritin levels below 60 µg/L, but without anemia (normal MCV), iron storage is moderate and its supplementation at low doses (i.e., up to 30 mg/day) may be considered, beginning with 16 week of pregnancy. In women with IDA (Hb < 11 g/dL and diminished concentration of ferritin), iron containing preparations should be applied before 16th week of pregnancy. Low doses of iron are recommended, administered orally for a prolonged time (taking into account the restricted pool of transporting and storage proteins) and change to more accessible preparations or increased doses of iron [12]. Iron containing preparations should be applied between meals, avoiding complex multimineral products, since many microelements (calcium, copper, zinc, manganese, iron) mutually restrict their absorption. The presence of ascorbic acid (vitamin C) and β-carotene (pro-vitamin A) increases, on the other hand, iron assimilation [1,18,24]. Noteworthy, many processes in iron metabolism are dependent upon the status of copper and zinc, so their supply (separately from iron) will be provided [24].

Lastly, it is well known that iron overload is dangerous due to increased blood viscosity and formation of toxic ROS that may lead to resistance to insulin and glucose intolerance, and gestational diabetes as a consequence, preeclampsia, placental infarction, premature delivery and developmental fetal defects [1,4,5,6,7,9,10,11,12,29]. As clinical studies in India have indicated, IDA in pregnant women is related to increased pro-oxidant components and reduced antioxidant enzymes and vitamins [35]. According to the review by Kumar et al., most of the animal and human studies found raised oxidative stress in anemic subjects, and further rinsing with iron administration [8]. It is also known that iron is essential not only for the host but also for pathogens (bacteria, fungi and parasite), and its excess may be conductive to infections and tissue inflammation, unfavorable change of gut microbiota (lactic acid bacteria with little requirement for iron are displaced by other more syderophilic and potential pathogenic microorganisms, e.g., *Escherichia coli* and *Salmonella* sp.) and intestinal inflammation and metabolic endotoxemia [4,16,36]. Although viruses do not require iron, the infected cell needs it for effective virion synthesis, then host iron overload also enhances viral progression [37]. Iron over-supplementation may be harmful, therefore numerous authors postulate the evaluation of the iron status of pregnant women for optimal supplementation [4,5,8,12,24].

## 5. Lactoferrin as a Multifunctional Protein

Lactoferrin is a well conserved, monomeric 80-kDa single polypeptide chain contained in most mammalian exocrine secretions, including milk, and also in the secondary granules of neutrophils [38,39,40,41]. LTF as one of the iron-binding transferrins can reversibly bind two iron ions (preferentially Fe^3+^) with high affinity (iron binding capacity of LTF is about 300-fold greater than that of TF) [42]. LTF, unlike TF, may sequestrate iron even at low pH, common in infected and inflamed tissues. Importantly, LTF is highly homologous across mammalian species, including bovine versus human. Bovine milk-derived LTF shares 70% amino acid sequence homology with human milk-derived LTF with very similar functionalities. At highest concentrations, LTF is present in body excretions and on mucous membranes of the eyes, gastrointestinal tract, respiratory and urogenital system. In these “gates of infection”, LTF fulfills a role as a very effective “guardian”, protecting the organism against viral, bacterial, fungal and parasitic infections [38,40,41,43]. Apart from direct destruction of pathogens it regulates activities of cells of innate and adaptive immunity, such as monocytes, macrophages, dendritic cells, neutrophils, eosinophils, mast cells, natural killer (NK) cells, B and T lymphocytes. LTF acts as immune regulator, in a manner dependent on the actual host’s immune status. So, it may activate immune cells for secretion of cytokines such as IL-1, IL-2, IFN, IL-8, IL-12, IL-15 and tumor necrosis factor (TNF)-α, increasing their anti-infectious and antitumor activities, and in parallel LTF-inducing IL-10 and transforming growth factor (TGF)-β, that lowers excessive reactivity of immune cells, may restrict inflammatory processes both in aseptic and septic inflammation [39,44,45]. The important aspect of LTF activity is regulation of physiological and pathological T helper (Th)1 and Th2 cellular immune responses, thereby limiting excessive inflammatory responses by changing its balance [39,44,46]. Repair of the Th1/Th2 imbalance by LTF is essential also in healing of autoimmune and allergic diseases. The preclinical and clinical data indicate that LTF as a pleiotropic immune modulator may be of therapeutic value in treatment of autoimmune disorders such as rheumatoid arthritis, colitis, encephalomyelitis/multiple sclerosis and psoriasis [47,48,49,50]. Interestingly, LTF also has the ability to lower intensity of hemolytic autoimmune anemia in animal model (New Zealand Black mice) by lowering recognition of self-antigens on erythrocytes [51].

LTF is also responsible for controlling of the toxic ROS activity, thus protecting cells from oxidative stress. By virtue of free iron sequestration, LTF controls the physiological balance of the production of ROS and the rate of elimination, which naturally buffers against direct oxidative cell injury [52]. At the molecular level LTF inhibits iron-dependent Fenton reaction and OH^•^ formation leading to lipid peroxidation and subsequent functional changes in biomolecules such as proteins, DNA and lipids [39]. Iron chelation is indeed the major mechanism by which LTF protects cells from oxidative injury; it reduces oxidative stress-induced apoptosis and attenuates mitochondrial dysfunction [53]. LTF increases also anti-oxidant enzymes gene expression, such as glutathione peroxidase (GPX), peroxiredoxins (PRDX), prostaglandin-endoperoxide synthase (PTGS) and superoxide dismutase (SOD) [54]. As described by Maneva et al., LTF may also be a regulator of the metabolic activity in erythrocytes by stimulation of glycolysis and antioxidative protection either in normal or in oxidative stress conditions [55]. Data from animal study indicate moreover that LTF application may counteract acute hemorrhagic anemia. LTF injected to rats with IDA resulting from regular blood losses normalized the iron metabolism, probably by stimulation of ferroxidase activity of ceruloplasmin, an essential copper-containing component in the iron metabolism [56].

Interestingly, LTF also has a prebiotic property protecting and stimulating growth of symbiotic bacteria in the gut and reproductive organs [40,57,58]. Supplementation of LTF also improves gut barrier function (by increasing expression of intestinal barrier proteins including ZO-1, occludin, claudin-1 and E-cadherin), preventing LPS translocation and following inflammation [59,60]. At high concentrations LTF is present in colostrum and mature milk, protecting suckling newborns against infection, oxidative stress and inflammation by stimulating gut microbiota growth, accelerating maturation of gut tissue and the immune system and protecting integrity of gut and central nervous system tissues. LTF fulfills, therefore, a particular significance in preterm newborns [61,62,63]. Other roles of LTF in human physiology encompass inhibition of tumor formation and metastasis, regulation of carbohydrates and lipid metabolism, promotion of bone formation and wound healing, and analgesic, anti-stress and hypotensive actions [64,65,66,67,68]. Recent data revealed the beneficial effects of oral LTF in vitamin D deficiency, such as reduction of inflammatory response, improvement of intestinal barrier function and normalization of colon microbiota [60]. Vitamin D deficiency will promote inflammation and dysbiosis in the gut, and is strongly associated with osteoporosis, autoimmune diseases and enteritis, as well as with increased hepcidin level and IDA [21]. LTF treatment also elevated the expression of colon vitamin D receptor (VDR) which mediates all biological actions of vitamin D and ensures intestinal homeostasis [60].

LTF indeed is a multifunctional protein and its many actions result from binding iron and interactions with numerous hosts’ molecules (cell receptors, glycosaminoglycans—GAGs, lysozyme, nucleic acids) as well as with various microbial molecules (e.g., cell receptors, nucleic acids, lipoteichoic acid—LTA, lipopolysaccharide—LPS). Endocytosed LTF can also bind to nuclear DNA and serve as a transcriptional factor [38,40,41,69].

## 6. Lactoferrin in Prophylaxis and Treatment of Anemia in Pregnancy

A considerable amount of evidence, especially during the last decade, demonstrated that LTF is a regulator of body iron metabolism [19,70,71,72]. LTF, endogenously released in the gut or delivered by diet, may act as iron supplier to epithelial cells of the intestine (Figure 2). The mechanism of this action is still unclear although it has been shown that iron saturated LTF facilitates iron transport by epithelial cells. LTF specific receptors on the apical side of the gut epithelium were found in sucklings and adults [69,73]. Microscopic examinations confirm that LTF molecules, saturated with Fe^3+^, bind to these receptors and penetrate the cells, subsequently releasing the transported iron. The expression of intestinal LTF receptors is regulated by the magnitude of cellular iron stores, and increases with its deficit. Higher number of receptors corresponds to higher uptake of iron. This interdependence has a particular significance during absorption of iron in newborns and youngest infants due to weakly developed mechanisms of classic regulation by means of membrane transporters DMT-1 and FPN. Therefore, LTF contained in maternal milk or artificial milk, ensures iron supply appropriate to the current needs in the intestine, protecting against deficit or excess [19,70,72,74].

The iron regulatory function of LTF has recently been confirmed in pregnant women in many clinical trials, including randomized ones (meta-analysis by Abu Hashim et al. [75]). Altogether 3367 women were monitored in various periods of pregnancy, both healthy and with chronic diseases, such as inherited thrombophilia, β-thalassemia, type 2 diabetes, hypertension, epilepsy or Crohn’s disease. The majority of trials were conducted in Italy and Egypt. In all trials native (iron saturated in 10%–20%) bovine LTF (BLTF) was used as oral tablets at daily doses of 25–250 mg that correspond merely to about 8–80 µg iron doses. In addition, in women with confirmed vaginal dysbiosis, thus in danger of premature delivery, BLTF was applied intravaginally. The control group was mostly treated orally with standard preparations of iron sulfate (corresponding to 156 mg of elementary iron). The therapy was conducted for 30–90 days or longer (from the end of first trimester to delivery) [76,77,78,79,80,81,82,83,84,85,86,87,88].

The efficacy of BLTF on iron status was comparable to, or even greater than that of inorganic iron formulas and was confirmed by analysis of iron status parameters: red blood cells (RBC) number, hematocrit (Ht) value, Hb level, sFtn level and total serum iron (TSI) concentration, total iron binding capability (TIBC) and other iron metabolism biomarkers. Additionally, in several studies a beneficial effect of BLTF supplementation on amelioration of the inflammatory state predisposing to premature delivery (decline in serum and cervical/vaginal fluid IL-6 level and serum hepcidin concentration) was demonstrated. Such a scenario is in agreement with results of in vitro tests demonstrating regulation by LTF of human macrophage function stimulated by LPS and IFN-γ. LTF changed the macrophage phenotype from proinflammatory to anti-inflammatory, by lowering synthesis of IL-1, IL-6 and hepcidin, increasing expression of FPN and ceruloplasmin and releasing iron, accumulated in body storage during inflammation [89,90].

Therefore, LTF exhibited a broad, more comprehensive, normalizing action on iron metabolism. On the other hand, the inorganic iron formulas did not affect, or even intensified, the inflammatory state, which additionally worsened body iron homeostasis [77,79,80,81,82,86]. In women receiving BLTF pregnancy lasts longer and newborns had higher birth weight [82,86]. Of importance, the patients treated with BLTF reported significantly fewer adverse effects (AEs) in the gastrointestinal tract and more readily applied the recommended treatment protocol than patients administered inorganic iron supplements (showing better compliance). These strong and threatening AEs of oral iron therapy are well known to affect the general wellbeing of women and, therefore, are the main reason for low compliance with this therapy. The results of the clinical trials with ID-/IDA-confirmed pregnant women are described in Table 1.

The role of LTF in iron absorption was also supported by several studies in non-pregnant women [79,91,92,93,94]. Human recombinant LTF produced in rice applied via a standard breakfast meal was as effective as ferrous sulfate in iron absorption by American young healthy women (*n* = 20). Iron absorption from both LTF and FeSO_4_ was negatively correlated to body iron status (sFtn concentration) [91]. BLTF intake was efficient for prevention of IDA among Japanese young health women (*n* = 10), young anemic women (*n* = 3) [93], and female long distance runners (*n* = 16) [92]. Anemia observed in athletes (sports anemia) is the result of, among other factors, lack of sufficient nourishment, hemolysis and breakdown of erythrocytes during heavy exercise, insufficient oxygen support and loss of iron in sweat, urine and stools. Especially, female athletes who are menstruating and controlling their weight are particularly prone to anemia. Oral BLTF administration (unlike ferrous sulfate) increased hematological parameters in 189 Italian non-pregnant women of child-bearing age affected by ID/IDA [79]. Oral BLTF, combined with recombinant human erythropoietin (rHuEPO), was also effective in 148 Italian advanced cancer patients (both sexes) undergoing chemotherapy [94]. Importantly, in all mentioned studies, LTF supplementation was shown to be safe and without any side effects.

## 7. Lactoferrin in Prophylaxis and Treatment of Inflammatory Complications in Pregnancy

**Figure 4:**
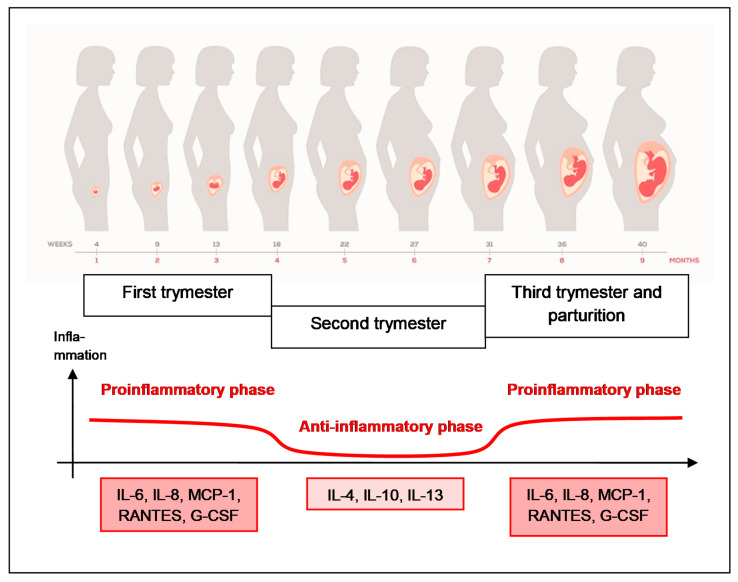
Immune response in normal pregnancy. Pregnancy may be divided into three immunologically different phases: the first trimester with inflammatory processes conditioned by proinflammatory cytokines and chemokines (IL-6, IL-8, MCP-1, RANTES, G-CSF), the second trimester with silencing of inflammatory reactions thanks to anti-inflammatory cytokines (IL-4, IL-10, IL-13) and the third trimester with the inflammation intensified again due to the activity of proinflammatory factors conditioning the proper course of delivery and childbirth.

During pregnancy the function of the immune system is not suppressed but is modulated which enables the proper course of implantation, development of embryo and fetus, and delivery [30,31]. Therefore, a sterile state of inflammation of a moderate intensity is inherent to normal pregnancy. Pregnancy may be divided into three immunologically different phases. The early period (the first trimester) is characterized by inflammatory processes conditioned by proinflammatory cytokines and chemokines (IL-6, IL-8, MCP-1, RANTES, G-CSF) usually associated with poor mental and physical wellbeing. During the next period (the second trimester) silencing of inflammatory reactions occurs thanks to anti-inflammatory cytokines (IL-4, IL-10, IL-13) that correlates with a rapid growth and development of the fetus and a positive frame of mind. In the last period of pregnancy (the third trimester) the inflammation intensifies again due to the activity of proinflammatory factors conditioning the proper course of delivery and childbirth (Figure 4). In spite of earlier theories suggesting immunosuppression during pregnancy (with a prevailing Th2-type immunity), the present view favors the existence of a precisely balanced, complex immune response in physiological pregnancy, tolerating a genetically foreign embryo/fetus and simultaneously protecting mother’s organism against infection. During pregnancy, a dynamic balance of the respective components of the immune response (Th1, Th2 and Th17 cells) is maintained with a particular role played by regulatory T (Treg) lymphocytes [30,31].

The state of physiological inflammation in pregnancy may be, however, pathologically elevated due to infections such as intra-amniotic infection and inflammation (IAI), infection of urinary and genital tracts and oral cavity, as well as additional inflammatory foci in connective tissue or gut or metabolic disturbances (obesity, diabetes, dyslipidemia, as elements of metabolic syndrome). Pregnant women are also in danger when subjected to invasive diagnostic procedures such as amniocentesis that potentially may evoke inflammation. ROS generated during iron supplementation constitute additional proinflammatory factors. An excessive inflammation, resulting from immune imbalance (imbalance between pro- and anti-inflammatory cytokines), creates a serious danger for pregnancy and may cause, among other factors, inhibition of embryo growth, preeclampsia, and premature delivery [31,95].

It can be, therefore, assumed that supplementation of diet with immunoregulatory iron-binding LTF will lead to restoration of iron status and immune homeostasis in pregnant woman. Given the protective (anti-infective and anti-inflammatory) role of endogenous LTF in all mucosal secretions including that of the genital tract, it is also important to emphasize the possible role of exogenous LTF in protection of woman and the fetus against infection and inflammation. Several studies on animals and pregnant women support the protective property of BLTF administered per os, intraperitoneally and intravaginally. In mice LPS-induced preterm delivery model intraperitoneal injection of LTF extended gestation by suppressing plasma proinflammatory IL-6 and TNF-α [96]. In turn, in rabbit preterm delivery models induced by bacteria, intravaginal LTF administration increased fetal survival and extended pregnancy by inhibition of matrix metalloproteinase activity [97]. In nine clinical trials 217 patients were investigated with diagnosed vaginal infection/inflammation, or liable to infection following invasive diagnostic procedures (amniocentesis) [80,81,95,98,99,100,101,102,103]. The administration of LTF normalized composition of vaginal microbiota, tonus of uterus cervix, silenced local inflammatory state, regulated the level of pro- and anti-inflammatory mediators, including cytokines, metalloproteinases and prostaglandins, and protected against oxidative stress. All these events correlated with general improvement of clinical state and prolongation of pregnancy to the physiological period. No AEs of the therapy were found. The results of clinical trial involving application of LTF in prophylaxis and treatment of infections and inflammations in pregnant women are described in Table 2.

## 8. Lactoferrin as a Diet Supplement

Bovine milk-derived lactoferrin was approved by the Food and Drug Administration (FDA) and the European Food Safety Authority (EFSA) as Generally Recognized as Safe (GRAS) for use as a food additive and dietary supplement many years ago [41,66,104,105]. The safety and tolerability of BLTF have been confirmed by numerous in vitro animal and human studies. Both acute, sub-chronic and chronic oral toxicological studies in rats orally administered with BLTF at doses of up to 2000 mg/kg b.w. showed that it is well tolerated with no AEs [105]. Carcinogenicity and genotoxicity of BLTF have not been detected [105]. A significant body of evidence from published intervention studies supports the safety of BLTF for humans, including infants and children. In the 30 clinical trials identified in infants (from preterm and term at birth—12 months) and in children (>12 months) and involving approximately 8000 subjects, no AEs or intolerances related to the administration of BLTF have been reported [106,107,108,109]. The identified studies, completed in both healthy and vulnerable infants and children, consistently report that BLTF is well tolerated in doses up to 450 mg/person/day in preterm and 2900 mg/person/day in term infants and up to 3000 mg/person/day in children.

**Figure 5:**
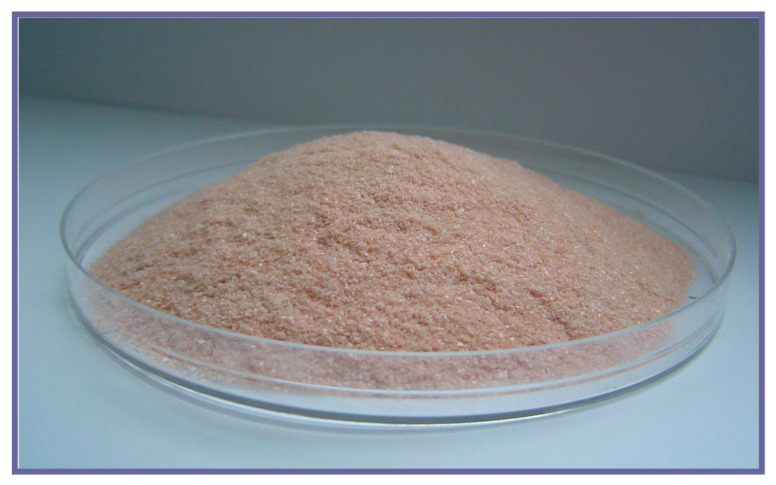
Lactoferrin (BLTF) isolated from bovine milk.

As such, BLTF has been in use in many countries’ diets for decades [105]. BLTF is isolated from cow milk by chromatography and freeze-drying giving a final product in the form of pink (salmon) powder, thanks to presence of Fe^3+^ (20–30% saturation) (Figure 5). The current global production of milk BLTF is estimated at 200 tons per year [105].

Oral management is safe, practical and the most desirable way of BLTF application, although other methods of administering BLTF include intravenous, intraperitoneal, intravaginal, intranasal, sublingual and on the wound [66]. BLTF demonstrates additive/synergistic activity with clinically used anti-infective (antiviral, antibacterial, antifungal and anti-parasite) compounds [43] and with probiotic strains of bacteria [57,58]. However, to the best of our knowledge there are no studies that report interaction of BLTF as a dietary supplement with any other drugs on the market.

It is worth mentioning some quality problems of BLTF supplements. The specification of Morinaga BLTF in GRAS was presented and discussed by Wakabayashi et al. [105], who ruled out possible negative effects of minute impurities in LPS (endotoxin) and milk ribonuclease (RNase) on its therapeutic efficacy in patients. On the other hand, others turned their attention to different efficacies of commercial BLTF preparations due to differences in their physicochemical properties [110]. For example, they reported that a BLTF preparation containing lactoperoxidase, as an impurity, showed less efficacy in the treatment of anemic patients in comparison with a homogenous lactoferrin preparation. In addition, effective BLTF capsules contained only the intact form of BLTF, contrary to the preparation with several degradation fragments. Antimicrobial activities of LTF may also depend on the degree of iron and other metal ions saturation and level of glycosylation [110].

Although, in comparison with other proteins, LTF is relatively resistant to high temperature and enzyme degradation, warming of LTF-containing preparations above 75 °C should be avoided. Ultra-high temperature (UHT) procedure (sterilization at 130–150 °C for a few seconds) seems to affect structural as well as certain biological properties (antibacterial) of both native and iron-saturated bovine lactoferrins [111]. Preparations containing LTF should be preferably taken between meals (weaker activity of digestive enzymes) or as an addition to calcium-rich dairy and vegetable products, as calcium ions stabilize the structure of the LTF molecule. Although LTF alone contains little iron (about 30–40 µg/100 mg LTF powder), if added to these products it will facilitate assimilation of their non-hem iron. LTF fulfils a similar role when naturally contained in diet and added to iron-containing diet supplements.

Orally administered LTF (as native peptide or partially digested) may reach the intestine where it interacts with the microbiota, epithelium and gut associated lymphoid tissue (GALT), activated by LTF immune cells or/and released cytokines reached by circulation in the major organs, affecting the function of other immunocompetent cells. In this way LTF may augment the immunity of all mucous membranes (eyes, gastrointestinal, urinary and reproductive tracts and airways) protecting against penetration of pathogens and development of excessive inflammation [58,112]. LTF, applied orally to rats, altered expression of numerous genes, such as genes coding for IL-1β, TNF-α, TNF-β and cellular receptors CD40L, CD80, IL-5R on peripheral blood leukocytes, similarly to the effects of intravenously administered LTF [113].

These results avert earlier suspicions with regard to systemic activity of orally administered LTF and may explain the efficacy of oral LTF in low doses (20–100 mg/person). In such cases LTF may interact with the mucous membrane of the oral cavity, peri-pharyngeal and intestinal lymphoid tissue. Treatment with LTF at 10, 40 or 50 mg doses led to stimulation of myelopoiesis and regulation (depending on the initial immune activity of the individuals) of spontaneous production of IL-6 and TNF-α in whole blood cultures of healthy volunteers [114,115], and applied at 20 mg to patients before surgery resulted in diminution of the post-operational decrease of immune reactivity, observed following surgery [116]. In addition, LTF applied per os in pregnant women at 25 mg daily dose (together with vitamins C, B12 and organic iron) improved hematological parameters, prolonged time of pregnancy and increased newborns’ weight [83].

## 9. Conclusions

Based on evidence reviewed above, the efficacy of LTF in the treatment of ID/IDA/AI is comparable to or better than supplementation with various inorganic and organic iron formulas. Multiple factors are likely to be responsible for such results considering that LTF not only facilitates delivery of iron to the specific receptors on the intestinal epithelium, but also presents a full spectrum of immunomodulatory functions and thus regulates systemic iron metabolism. In particular, by inhibition of inflammatory processes [39,45], LTF restores equilibrium of FPN and hepcidin, key “players” in iron metabolism [45,71,110,117]. The efficacy of LTF in the treatment of anemia in pregnancy strongly suggests that absolute iron deficiency is not the sole reason for occurrence of anemia, but rather iron functional immobilization and lack of its availability for cellular metabolic function and hematopoiesis. Such a state results from chronic inflammation (anemia of inflammation), in particular in women with chronic diseases (inherited thrombophilia or type 2 diabetes). Substantial improvement of hematologic parameters following supplementation with LTF resulted in a decrease of proinflammatory IL-6 levels, which, in turn, led to modulation of hepcidin concentrations and FPN activity [117]. In this way, following the silencing of the inflammatory response, LTF normalizes iron homeostasis [45,71,89,90].

The literature data conclude that iron supplementation in ID/IDA treatment is not a priority, but rather restoration of systemic homeostasis by correct diagnosis and appropriate treatment of inflammatory states of various origin. Of note, iron supplementation in anemia is of low efficiency and even deleterious because of increase in ROS formation and promotion of inflammation and pathogen growth. Thus, benefits of iron supplementation may be only achieved after normalization of overall iron metabolism. Application of LTF may perfectly fit the procedure of correction of iron metabolism. Importantly, LTF may be applied to prevent and treat anemia without the risk of toxicity often encountered with inorganic iron supplementation.

In parallel, pregnant women taking LTF as a source of iron should benefit from other actions of LTF. These encompass prebiotic properties of LTF in the gastrointestinal and genital tract, protection of the mucous layer of the gastrointestinal tract, promotion of bone formation and wound healing, normalization of sugar and lipid metabolism, as well as hypotensive, antistress and analgesic actions.

## Figures and Tables

**Table 1:** Clinical studies with BLTF in therapy of pregnant women suffering from ID/IDA.

Type of Study, Number of Participants, Country	Type of BLTF, Dose, Mode and Time of BLTF Application	Clinical/Laboratory Effects	References
Randomized study; *n* = 300 (pregnant women at 12–31 weeks of pregnancy suffering from ID or IDA); Italy	BLTF (Lf100^®^, Dicofarm, Italy) p.o., 100 mg/person/day b.i.d. (daily doses 200 mg/person, corresponded to 70–84 µg of iron ions/day), every day for 4 weeks Ferrous sulfate FeSO_4_ (520 mg/person/day corresponded to 156 mg of Fe^2+^) as control No supplement as additional control	Hb↑, TSI↑ No gastrointestinal EAs (abdominal pain, constipation) Compliance↑	[76]
*n* = 143 (pregnant women suffering from ID or IDA); Italy	BLTF ^#^ p.o., 100 mg/person/day b.i.d. (daily doses 200 mg/person), every day for 30 days FeSO_4_ 520 mg as control No supplement as additional control	RBC↑, Hb↑, TSI↑, sFtn↑	[77]
*n* = 5 (pregnant women suffering from ID or IDA); Italy	BLTF ^#^ p.o., 100 mg/person/day b.i.d. (daily doses 200 mg/person), every day for 30 days FeSO_4_ 520 mg as control	RBC↑, Hb↑, TSI↑, sFtn↑ Serum IL-6↓	[77]
Prospective, one-left, randomized, open-label, phase IV study; *n* = 75 (pregnant women in their third trimester of pregnancy suffering from ID or IDA); Italy	BLTF (Lattoglobina^®^, Grunenthal-Formenti, Italy) p.o., 100 mg/person/day b.i.d. (daily doses 200 mg/person), every day for 30 days FeSO_4_ 520 mg as control No supplement as additional control	RBC↑, Ht↑, Hb↑, TSI↑, sFtn↑ Serum IL-6↓ Serum prohepcidin↑ (as a parameter of normalization of iron metabolism) Gastrointestinal EAs↓ Compliance↑	[79]
Open-label, cohort and subcohort study; *n* = 161 (pregnant women at their second and third trimester of gestation with ID/IDA) in cohort and *n* = 11 (pregnant women with preterm delivery threat not related to cervical and vaginal infections) in subcohort; Italy	BLTF (Lattoglobina^®^, Grunenthal-Formenti, Italy) p.o., 100 mg/person/day b.i.d. (daily doses 200 mg/person), everyday for 4 weeks until delivery (in cohort); additionaly BLTF (the same preparation in vaginal tablets) intravaginally; 100 mg t.i.d. (daily doses 300 mg/person) for 4 weeks of gestation, no longer than 37th week of gestation (in subcohort)	In cohort: RBC↑, Hb↑, TSI↑, sFtn↑ Serum IL-6↓ In subcohort: RBC↑, Hb↑, TSI↑, sFtn↑ Serum and cervicovaginal IL-6↓ Cervicovaginal PGF2α ↓ Suppressed uterine contractility, block further shortening of cervical length and the increase of fetal fibronectin; prolonging the length of pregnancy; the deliveries between 37–38th week of gestation No maternal and fetal/newborn AEs	[81]
Interventional, non-randomized, one-left study; *n* = 295 (pregnant women affected by HT enrolled between the 6th and 8th weeks of pregnancy suffering from ID/IDA); Italy	BLTF (Lattoglobina^®^ or Isoferine^®^ Grunenthal-Formenti, Italy) p.o., 100 mg/person/day b.i.d. (daily doses 200 mg/person), every day until delivery FeSO_4_ 520 mg as control	RBC↑, Hb↑, TSI↑, sFtn↑ Serum IL-6↓ Gastrointestinal EAs↓ Well-being fetus and newborns in both groups. 5 miscarriages in ferrous sulfate group and no miscarriages in BLTF group	[82]
Prospective, one-left, randomized, controlled, double-blind study; *n* = 100 (pregnant women in their second and third trimester of pregnancy suffering from IDA); Italy	BLTF (Elleffe^®^, Dicofarm, Italy) p.o., 100 mg/person/day b.i.d. (daily doses 200 mg/person), every day for 30 days FeSO_4_ 520 mg as control	Hb↑, TSI↑, sFtn↑ TIBC↓ Gastrointestinal EAs (abdominal pain, constipation)↓	[78]
Open-label, pilot study; *n* = 21 (26–32 weeks pregnant women, suffering from IDA, at risk of preterm delivery); Italy	BLTF (Lattoferrina^®^, AG Pharma, Italy) p.o., 100 mg/person/day b.i.d. (daily doses 200 mg/person), every day for 1 month FeSO_4_ 520 mg as control	Normalization of vaginal microbiota (vaginal infection disappearance) Cervicovaginal IL-6↓ Cervical length and funneling (in the ultrasound data) did not change at follow-up after 10 and 30 days; all women had term delivery	[80]
Non-randomized, multicenter study; *n* = 1143 (0–39 weeks pregnant women, suffering from IDA); Italy	BLTF (Lafergin^®^, Avantgarde, Gruppo Sigma-Tau), a dietary multicomponent based on: 60 mg of NaFe^3+^-EDTA (corresponding to 7.8 mg of Fe^3+^), 25 mg of BLTF, 0.002 mg of vitamin B12 and 70 mg of vitamin C; daily doses 1 tablet/person from 12 week of gestation to the end of gestation Liposomal iron or ferrous sulfate as controls	Hb↑, sFtn↑ Higher mean birth weight of newborns and longer duration of pregnancy Gastrointestinal AEs (abdominal cramps, constipation, diarrhea)↓	[83]
Interventional, one-left study; *n* = 198 (pregnant and non-pregnant women suffering from IDA and AI) *n* = 70 (pregnant women affected by HT) *n* = 79 (non-pregnant women affected by HT) *n* = 20 (β-thalassemic pregnant women) *n* = 9 (β-thalassemic non-pregnant women) *n* = 20 (pregnant women affected by diabetes type 2, epilepsy, Crohn’s disease, hypertension); Italy	BLTF (Lattoglobina^®^, Grunenthal-Formenti, Italy) p.o., 100 mg/person/day b.i.d. (daily doses 200 mg/person), every day to the end of gestation FeSO_4_ 329.7 mg = 105 mg of Fe^2+^ as control	In pregnant and non-pregnant women: RBC↑, Hb↑, TSI↑, sFtn↑ Serum IL-6↓ Serum hepcidin ↓	[86]
Prospective, randomized, parallel-group, multicenter study; *n* = 300 (pregnant women in their second trimester of pregnancy suffering from IDA); Egypt	BLTF (Jarrow Formulas, Egypt) p.o., 250 mg once daily for 8 consecutive weeks Ferrous sulfate 150 mg or ferrous fumarate 350 mg as control	Hb↑ Gastrointestinal AEs (gastric upset, abdominal pain, vomiting, constipation and dark stool) ↓Compliance↑	[85]
One-left study, double-blind study; *n* = 188 (pregnant women at their second trimester of gestation suffering from IDA), Egypt	BLTF (Pravatin^®^, Hygint, Egypt), p.o., 100 mg/person/day b.i.d. (daily doses 200 mg/person) for 8 weeks FeSO_4_ 150 mg and folic acid 0.5 mg as control	Hb↑, sFtn↑ Gastrointestinal AEs (abdominal pain, constipation, nausea, vomiting)↓	[87]
Interventional, randomized, parallel-group, single-left study; *n* = 120 (pregnant women, suffering from ID/IDA); Egypt	BLTF *^#^ p.o., 100 mg b.i.d. (daily doses 200 mg/person) everyday for 4 weeks plus health education Total dose infusion (TDI) of low-molecular weight iron dextran as control group	Clinical improvement Hb→ MCV↑ MHC↑ TSI↑, sFtn↑	[88]
Prospective, multi-left study; *n* = 406 (pregnant women at different gestational ages, suffering from ID/IDA); Romania	BLTF ^#^ p.o., 100 mg b.i.d. (daily doses 200 mg/person) everyday for 90 days No control group	Correction of the iron deficiency in ~91% of patients with ID and patients with IDA Hb↑, TSI↑ Gastrointestinal AEs↓ Compliance↑ Satisfactory the APGAR score and the birth weight of the newborns	[84]

AEs—adverse events; AI—anemia of inflammation; b.i.d.—bis in die, two times a day; BLTF—bovine lactoferrin; Hb—hemoglobin; Ht—hematocrit; HT—hereditary thrombophilia; LTF—lactoferrin; ID—iron deficiency; IDA—iron deficiency anemia; MCH—mean corpuscular hemoglobin; MCV—mean corpuscular volume; PGF2α—prostaglandin F2α; RBC—red blood cells; sFtn—serum ferritin; TIBC—total iron-binding capacity; TSI—total serum iron; ^#^ the authors did not depict what kind of LTF preparation was used; * only abstract was available.

**Table 2:** Clinical studies with BLTF in prophylaxis and therapy of pregnant women suffering from infection and inflammation.

Type of Study, Number of Participants, Country	Type of LTF, Dose, Mode and Time of LTF Application	Clinical/Laboratory Effects	References
Randomized, open-label study; *n* = 60 (pregnant women undergoing genetic amniocentesis at the 16th gestational week); Italy	BLTF (Difesan^®^, Progine Farmaceutici, Firenze, Italy), 300 mg in vaginal tablet, once 4 h or 12 h prior amniocentesis	Amniotic IL-6↓	[95]
		Decreased levels of amniotic proinflammatory mediators: IL-9, IL-15, IFN-γ, IP-10, TNF-α, IL-1α, MCP-3, IL-2RA, IL-12p40, IFN-α2, IL-2, IL-4, eotaxin, PDGF-BB, RANTES, IL-18, MIF ↓ Increased levels of amniotic anti-inflammatory mediators: IL-17, FGF-b, G-CSF, GM-CSF, MCP-1, IL-3, SDF-1α↑	[101]
	BLTF (Difesan^®^, Progine Farmaceutici, Firenze, Italy), 300 mg in vaginal tablet, once 4 h or 12 h prior amniocentesis In vitro test on antioxidant effect of LTF: human monocytic U937 cell line was treated with 50 μg/mL LTF ^#^ for 4 h or 12 h	Decreased oxidative stress in vivo: Amniotic TBARS concentration↓ Amniotic TAS↑ Decreased oxidative stress in vitro: TBARS concentration↓	[103]
Prospective, randomized study; *n* = 111 (pregnant women undergoing genetic amniocentesis at the 16–18th gestational week); Italy	BLTF (Difesan^®^, Progine Farmaceutici, Firenze, Italy), 300 mg in vaginal tablet, once 4 h prior amniocentesis	Regulation of the inflammatory markers in the amniotic fluid: PGE2, MMP-9 and TIMP-1 (inhibitor of MMP-1) ↓ MMP-2 ↑ TIMP-2 (inhibitor of MMP-2) →	[99]
Case report; 38-year-old multiparous women with 3 preterm premature rupture of membrane, diagnosed as having refractory vaginitis (*Streptococcus* group B and *Staphylococcus*), not cured with estriol and antibiotics; Japan	BLTF (NRL Pharma, Kawasaki, Japan) intravaginal, 150 mg/day and p.o. 700 mg/day for 41 weeks (13 weeks before pregnancy and 38 weeks after, until delivery)	Appearance of *Lactobacillus* in vaginal flora, patient achieved pregnancy 3 months later and delivered a health infant After the delivery LF application was discontinued and 1 and 3 months after no *Lactobacillus* was detected in vaginal discharge cultures	[98]
*n* = 6 (5 pregnant and 1 nonpregnant women with a history of multiple pregnancy losses or preterm delivery and refractory bacterial vaginosis *E. coli*, *Enterococcus*, *Gardnerella vaginalis*, *Staphylococcus*); Japan	BLTF (NRL Pharma, Kawasaki, Japan) intravaginal, 150 mg/day and p.o. 700 mg/day, start before pregnancy or from 11–21th gestational week until delivery	Normalization of vaginal flora (appearance and gradual predominance of *Lactobacillus*) Patients achieved pregnancy and delivered at term No AEs in mothers and newborns	[102]
Open-label cohort study; *n* = 7 (pregnant women asymptomatically affected by *Chlamydia trachomatis* and showing high concentration of IL-6 in cervical fluids; Italy	BLTF (Morinaga Milk Ind.) intravaginal, 100 mg every 8 h (daily doses 300 mg/person) for 30 days In vitro test on anti-chlamydial effect of LTF: human epithelial HeLa-229 cell line was treated with 100 μg/mL BLTF	In vivo test: 6 out of 7 cervical specimens negative to *C. trachomatis* IL-6 in cervical fluids ↓ Patients achieved pregnancy and delivered at term No maternal and neonatal AEs In vitro test: inhibitory effect of BLF on *C. trachomatis* entry to cells, decrease of IL-6 and IL-8 levels induced by infection with *C. trachomatis*	[100]

AEs—adverse events; BLTF—bovine lactoferrin; FGFb—basic fibroblast growth factor; G-CSF—granulocyte colony-stimulating factor; GM-CSF—granulocyte-macrophage colony-stimulating factor; IFN—interferon; IL –interleukin; IP-10—interferon inducible protein; LTF—lactoferrin; MCP-1—monocyte chemoattractant protein-1; MIF—macrophage migration inhibitory factor; MMP-2—matrix metalloproteinase-2; MMP-9—matrix metalloproteinase-9; OSI—oxidative stress index; PDGF—platelet-derived growth factor; PGE2—prostaglandin E2; RANTES—regulated on activation normal T cells expressed and secreted; SDF-1α—stromal cell-derived factor 1α; TAS—total antioxidant status (expressed in Trolox equivalents or OSI); TBARS—thio-barbituric acid reactive substances (expressed as malondialdehyde—MDA equivalents); TIMP-1—tissue inhibitor of metalloproteinase-1; TIMP-2—tissue inhibitor of metalloproteinase; TNFα—tumor necrosis factor α; # the authors not depicted what kind of LTF preparation was used.

## Data Availability

No new data were created or analyzed in this study. Data sharing is not applicable to this article.
